# Icariin Inhibits AMPK-Dependent Autophagy and Adipogenesis in Adipocytes *In vitro* and in a Model of Graves' Orbitopathy *In vivo*

**DOI:** 10.3389/fphys.2017.00045

**Published:** 2017-02-13

**Authors:** Hong Li, Yifei Yuan, Yali Zhang, Xia Zhang, Long Gao, Rongjuan Xu

**Affiliations:** ^1^Department of Endocrinology, Longhua Hospital Shanghai University of Traditional Chinese MedicineShanghai, China; ^2^Department of Ophthalmology, Eye and ENT Hospital of Fudan UniversityShanghai, China; ^3^Institute of Spleen and Stomach Disease, Longhua Hospital Shanghai University of Traditional Chinese MedicineShanghai, China

**Keywords:** Graves' orbitopathy, autophagy, adipogenesis, thyroid stimulating hormone receptor, icariin

## Abstract

Graves' orbitopathy (GO), an extrathyroidal manifestation of Graves' disease, is an inflammatory autoimmune disorder of the orbit that involves the differentiation of precursor cells into mature adipocytes and retro-orbital adipose tissue accumulation. Here, we examined the involvement of autophagy in adipogenesis and explored the effects of icariin, a flavonoid isolated from the genus *Epimedium* with a wide range of biological and pharmacological effects, on autophagy and adipogenesis in 3T3-L1 preadipocytes and in a mouse model of GO. Microscopic examination of autophagosome formation and lipid droplet accumulation by Oil Red O staining, and western blot assessment of autophagic markers in the presence of the autophagy inhibitors Asn and 3-MA showed that autophagy is essential for adipogenesis. Icariin inhibited the differentiation of preadipocytes into mature adipocytes by suppressing autophagy, and these effects were mediated by the inhibition of AMPK/mTOR pathway activation. In a mouse model of thyroid stimulating hormone receptor induced GO, icariin reduced orbital muscle adipose tissue expansion and lipid droplet accumulation by inhibiting AMPK/mTOR mediated autophagy. Collectively, these results reveal a potential mechanism underlying the protective effects of icariin against autophagy induced adipogenesis and suggest that icariin could be developed as a new therapeutic candidate for the prevention and treatment of GO.

## Introduction

Graves' disease is an autoimmune disease characterized by the binding of autoantibodies to the thyrotropin receptor on follicular cells of the thyroid gland, resulting in the activation and enlargement of the thyroid gland (Davies et al., 2005). Graves' orbitopathy (GO) is a complication of Graves' disease whose features include upper eyelid retraction, edema, periorbital tissue erythema, and proptosis, which in a fraction of patients can progress to optic neuropathy, thus compromising sight (Garrity and Bahn, 2006). Severe GO is associated with increased formation of connective/fatty tissues within the bony orbit, and orbital adipose tissue in GO contains increased amounts of preadipocytes that can differentiate into adipocytes (Crisp et al., 2000).

Adipogenesis, the differentiation of pre-adipocytes into mature adipocytes, is characterized by the activation of transcription factors such as peroxisome proliferator-activated receptor γ, members of the CCAAT/enhancer-binding protein family, and sterol regulatory element binding protein-1 (Tang and Lane, 2012). These factors modulate the expression of genes essential for adipocyte differentiation, which is characterized by the formation of lipid droplets in the cytosol for energy storage (Zhang et al., 2012). Adipose tissue formation involves the process of autophagy, in which cytoplasmic materials are packaged into double membrane structures known as autophagosomes and targeted for lysosomal degradation (Singh et al., 2009). Autophagy is regulated by two factors, the mammalian target of rapamycin (mTOR) and adenosine monophosphate-activated protein kinase (AMPK), a serine-threonine kinase that functions as a metabolic sensor and activates autophagy in response to low energy levels (Kubli and Gustafsson, 2014). AMPK is activated by changes in the availability of ATP and AMP in the cytosol, and AMPK activation leads to the inactivation of energy consuming pathways and the activation of energy producing pathways, thus restoring energy homeostasis (Hardie et al., 2012).

Icariin, a flavonoid isolated from several species of plants in the genus *Epimedium*, has a wide range of effects (Arief et al., 2015) including cardioprotective (Ke et al., 2015), vasodilatory (Xu and Huang, 2007), and immunomodulatory effects (Liang et al., 1997; Tang et al., 2015). Icariin was shown to promote proliferation, migration, and capillary tube formation in bone marrow derived endothelial progenitor cells (BM-EPCs), suppressing hydrogen peroxide induced apoptosis and autophagy by activating mTOR and suppressing p38 MAPK and ERK1/2 signaling.

In the present study, we examined the effects of icariin on autophagy-mediated adipogenesis in preadipocytes *in vitro* and in a mouse model of GO *in vivo* and explored the underlying mechanisms.

## Materials and methods

### Reagents

Icariin, L-asparagine (Asn), 3-methyladenine (3-MA), Oil Red O, MTT assay kit, uranyl acetate/lead citrate, and MDC were purchased from Sigma-Aldrich, Inc. (St. Louis, MO, USA). Dulbecco's modified Eagle's medium (DMEM), fetal bovine serum (FBS), penicillin, adipocyte differentiation medium, and gentamycin were purchased from Hyclone Laboratories, Inc. (Logan, UT, USA). The Annexin V-FITC apoptosis detection kit was purchased from BD Biosciences (Franklin Lakes, NJ, USA). Anti-mTOR, anti-p-mTOR, anti-beclin-1, anti-AMPK, anti-p-AMPK, anti-p62, anti-LC3, and anti-β-actin antibodies were obtained from Santa Cruz Biotechnology (Santa Cruz, CA, USA).

### Animals and ethical statement

Female BALB/c mice (8–10 weeks old) were obtained from the Shanghai SLAC Laboratory Animal Co., Ltd (Shanghai, China). All animals were treated in accordance with the Guide for the Care and Use of Laboratory Animals, and all experiments were approved and performed by the Longhua Hospital Ethics Committee of China.

### Adipocyte differentiation

3T3-L1 preadipocytes (Purchased from Procell Life Science Co. Ltd., Wuhan, China) were cultured and maintained in 1 × DMEM supplemented with 10% FBS and antibiotics (500 μg/mL penicillin and 500 μg/mL streptomycin; maintenance medium) at 37 °C in a humidified atmosphere with 5% CO_2_. For adipocyte differentiation, 100% confluent 3T3-L1 cells were incubated for 2 days to induce complete cell cycle arrest and then incubated in differentiation medium (DM; maintenance medium supplemented with 160 nM insulin, 250 nM dexamethasone, and 0.5 mM 1-methyl-3-isobutylxanthine; day 0) to begin clonal expansion. After 2 days, the cells were further incubated in maintenance medium supplemented with 160 nM insulin, and subsequently incubated in the maintenance medium after another 2 days. Then, half of the cell medium was replaced by fresh maintenance medium every 2 days until the cells were completely differentiated (14 days). To determine the effect of icariin on adipocyte differentiation, 5 μM of icariin were added to the medium before cells were cultured in DM. To assess the effect of autophagy on adipocyte differentiation, two autophagic inhibitors were added to medium (Asn, an inhibitor of autophagosome-lysosome fusion at 250 mM or 3-MA, an inhibitor of phosphoinositide 3 kinase that specifically inhibits autophagosome formation at 10 mM) before culture of cells in DM. All treatments were added to the medium after cells reached confluence and before the addition of adipocyte DM.

### Cell viability assay

3T3-L1 cells were seeded in 24-well culture plates at a density of 1 × 10^5^ cells/well and treated with different concentrations of icariin (0, 1, 2.5, 5, or 10 μM) for 48 h. After treatment, cells were washed, incubated with 5 mg/ml MTT solution for 4 h at 37°C, and the resulting precipitate was solubilized in ice-cold isopropanol. The absorbance of the dye was measured at 560 nm, with background subtraction at 630 nm, with a microplate reader (EL 340 Biokinetics Reader; Bio-Tek Instruments, Winooski, VT, USA).

### Apoptosis assay

The effect of icariin on preadipocyte 3T3-L1 apoptosis was evaluated using an Annexin V/FITC kit. Cells were washed with isotonic phosphate buffered saline (PBS) and then incubated in serum-free DMEM in the presence of different concentrations of icariin for 6 or 24 h, after which the apoptosis assay was performed according to the procedure recommended by the manufacturer. For flow cytometric analysis, 1 × 10^4^ cells were excited at 488 nm, and emission was measured at 530 and 584 nm to assess FITC and propidium iodide fluorescence, respectively.

### Oil Red O staining and quantification

Cells were washed twice with 1 × PBS, fixed in 3.7% formaldehyde for 10 min, and then washed three times with cold water. Cells were stained in the Oil Red O working solution (6:4, 0.6% Oil Red O dye in isopropanol:water) for 30 min at 25°C and washed three times with water. Staining was visualized by bright-field microscopy, and Oil Red O dyes extracted from the cells in isopropanol solution containing 4% Nonidet P-40 were quantified at a wavelength of 520 nm.

### Assessment of autophagosome formation by transmission electron microscopy (TEM)

The 3T3-L1 cells were post-fixed in osmium tetroxide (OsO_4_) and embedded in Epon. Sections were then stained with uranyl acetate/lead citrate and viewed with a JEM1230 transmission electron microscope (JEOL, Tokyo, Japan). The 3T3-L1 cells on cover slips were stained with MDC and visualized with an SP5 confocal system (Leica, Solms, German) with excitation and emission filters of 380 nm and 525 nm, respectively.

### Western blot analysis

3T3-L1 cells were collected, washed twice with ice-cold 1 × PBS, and lysed in RIPA buffer (50 mM Tris-HCl, pH 7.2, 150 mM NaCl, 1% NP-40, 0.5% sodium deoxycholate, and 0.1% SDS) containing protease inhibitors (1 mM phenymethylsulfonyl fluoride, 1 μg/mL aprotinin, 1 μg/mL leupeptin) on ice for 1 h. Cell lysates were centrifuged at 12,000 rpm for 15 min at 4°C. The supernatant was then collected for SDS-PAGE analysis. β-actin was used as a loading control. Samples (40 μg total protein) were separated on SDS-PAGE and transferred to nitrocellulose membranes (Millipore, USA). After blocking in 5% non-fat milk for 1 h, the membranes were incubated with primary antibodies against mTOR (1:1000), p-mTOR (1:1000), beclin-1 (1:200), AMPK (1:200), p-AMPK (1:200), p622 (1:400), LC3 (1:400), and β-actin (1:1000), respectively, at 4°C overnight. After washing, the membranes were incubated with horseradish peroxidase-conjugated secondary antibodies for 1 h at room temperature. Signals were detected using an ECL detection system (GE Healthcare, USA) and analyzed by ImageJ 1.42q software (National Institutes of Health).

### Fluorescence microscopy

3T3-L1 cells were seeded into 24-well plates (5 × 10^4^ cells/well) with 10% FBS at 37°C in an atmosphere containing 5% CO_2_. When 3T3-L1 cells reached 70% confluence, lentivirus with GFP plasmid (Invitrogen) was added to the wells based on a multiplicity of infection (MOI) of 20. The medium was replaced at 24 h after infection and the fluorescence intensity was measured after 96 h. Non-infected 3T3-L1 cells were used as negative controls. GFP-labeled 3T3-L1 cells were cultured in control medium (FM) or DM for 0–14 days with various treatments. Treated cells were fixed with 4% paraformaldehyde, and viewed with an LSM 510 META confocal laser microscope (Carl Zeiss Ltd, Germany).

### Animal experiments

Induction of Graves' disease with an adenovirus expressing the human thyroid stimulating hormone receptor (TSHR) A-subunit (Ad-TSHR289) was performed as described previously, with the null adenovirus as the control (Liu et al., 2016). In brief, Ad-TSHR289 and a control adenovirus were propagated in HEK293 cells and purified via ion-exchange column chromatography. The viral particle concentration was determined by measuring the absorbance at 260 nm. Immunization with recombinant adenovirus in mice was performed as described previously (Liu et al., 2016). Briefly, one group of mice was injected with the null adenovirus intramuscularly and the other group was injected with Ad-TSHR289 in 50 μL of PBS containing 10^9^ particles. The two groups of mice were divided into two sub-groups, one was treated with 10 mg/kg icariin and the other group was treated with PBS intragastrically every day for 4 weeks. Immunizations were performed after 1 week of icariin or PBS intragastric treatment. Each animal was injected three times at 3-week intervals. Mice were sacrificed 4 weeks after the third immunization. Then, adipose tissue and orbital muscle in orbital sections were separated for further study.

### Histological examination

Tissues from orbital sections were fixed in 10% buffered formalin and paraffin embedded, and serial sections were stained with hematoxylin and eosin (HE). Sections were analyzed with an Olympus Cue-2 image analysis system connected to an Olympus compound microscope.

### Statistical analysis

All data are presented as the mean ± *SD*. Differences between treatment groups were analyzed using one-way ANOVA with *post hoc* tests. In all cases, *p* < 0.05 was considered to be statistically significant.

## Results

### Effect of icariin on cell viability and apoptosis

To assess the effects of icariin on cell viability and apoptosis, 3T3-L1 preadipocytes were treated with increasing concentrations of icariin (0–10 μM; Figure S1). Cell viability and apoptosis were assessed by MTT assay and Annexin V/FITC double staining. The results showed that there was no significant difference between cell numbers with or without icariin indicating that it had no effect on cell viability (Figure S1A) or apoptosis rates (Figure S1B).

### Adipocyte differentiation of 3T3-L1 cells

The level of differentiation was determined by incubating 3T3-L1 preadipocytes in adipocyte DM for 0–14 days and then assessing the changes by bright-field microscopy (Figure S2A) and Oil Red O staining (Figure S2B). Figure S2 shows representative images of cells at different time points, illustrating the appearance of lipid droplets on day 7. A significant increase in Abs 500 nm confirmed differentiation into adipocytes at day 14 (Figure S2C). These results indicate the successful differentiation of 3T3-L1 cells into adipocytes.

### Adipocyte differentiation is dependent on autophagy

**Figure 1:**
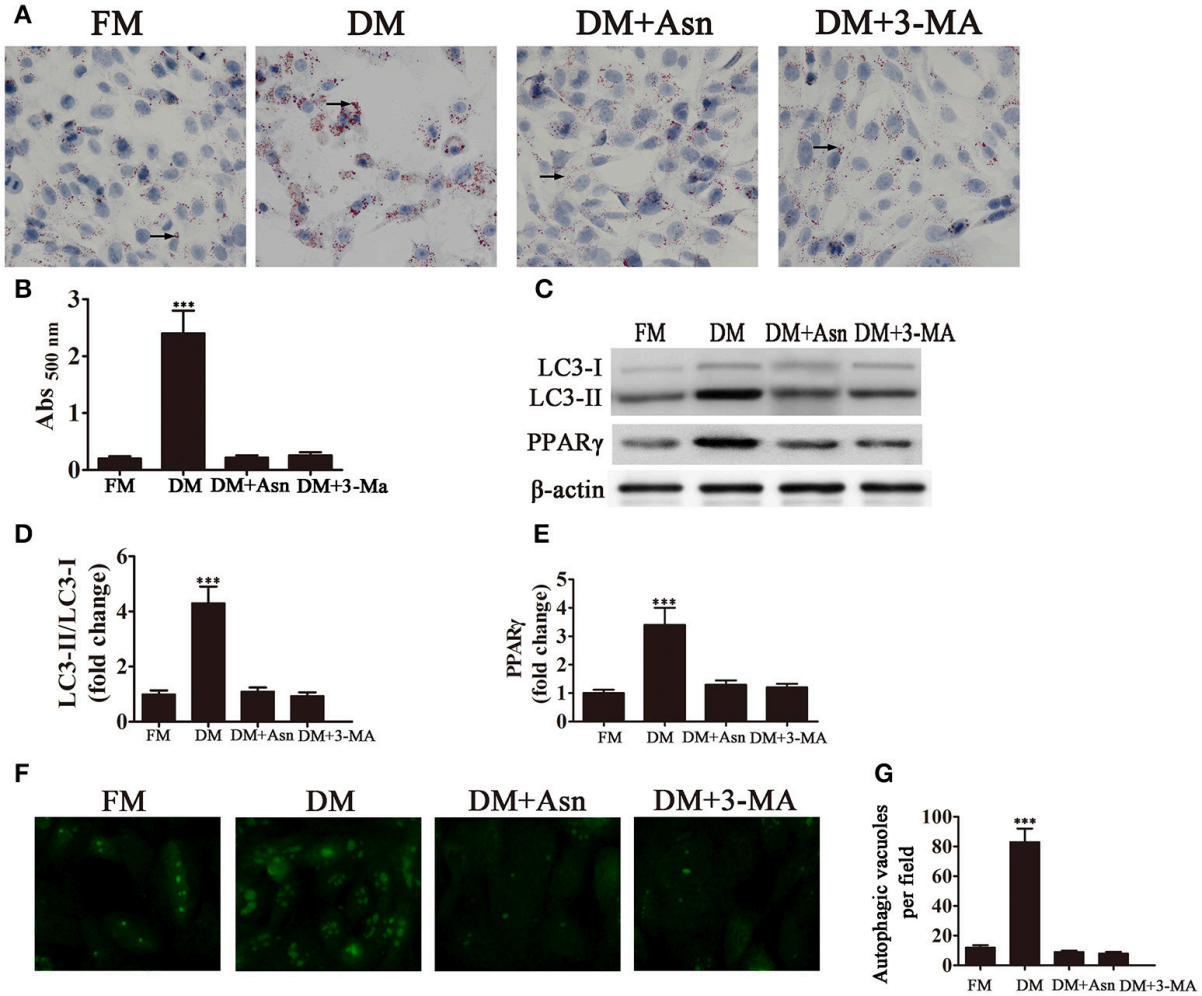
**Autophagy is necessary for adipocyte differentiation**. 3T3-L1 cells were exposed to control medium (FM) or adipocyte differentiation medium (DM) in the presence or absence of the autophagy inhibitors Asn and 3-MA for 14 days. **(A)** Oil Red O staining shows the effect of autophagy on lipid droplet formation. **(B)** Quantification of intracellular lipids of 3T3-L1 cells by spectrophotometry. Data represent the mean ± *SD*. ^***^P < 0.001 vs. the untreated control (*n* = 5). **(C)** Western blot analysis of the expression of the autophagy markers LC3-I, LC3-II and PPARγ with β-actin as the loading control. **(D,E)** The LC3-II/LC3-I ratio and relative PPARγ expression were quantified by densitometric scanning and graphed. Data indicate the mean ± *SD* (*n* = 5). ^***^P < 0.001 vs. control. **(F)** Representative fluorescence microscopy images of GFP-LC3 transfected cells treated as indicated. **(G)** The number of autophagosomes was counted in 10 random fields. Data represent the mean ± *SD*. ^***^P < 0.001 vs. the untreated control (*n* = 10). FM, control medium; DM, differentiation medium.

To determine the role of autophagy in the differentiation of preadipocytes into adipocytes, cells were exposed to DM for 14 days and treated with or without the autophagy inhibitors Asn and 3-MA. Oil Red O staining showed that treatment with Asn or 3-MA partially inhibited adipocyte differentiation. Western blot analysis confirmed that Asn or 3-MA treatment inhibited PPARγ expression induced by DM (Figures 1A–C,E). The role of autophagy in adipocyte differentiation was confirmed by western blot assessment of autophagic markers. The cytosolic form of the autophagic marker LC3, LC3-I, is conjugated to phosphatidylethanolamine to form LC3-II, which is recruited to autophagosomal membranes and degraded after fusion with the lysosome (Tanida et al., 2008). The ratio of LC3-II to LC3-I is therefore used as a marker of autophagy. Figures 1C,D shows that the LC3-II:LC3-I ratio increased significantly by approximately 4-fold in 3T3-L1 cells cultured in DM, whereas co-treatment with Asn or 3-MA restored the LC3-II:LC3-I ratio to pre-differentiation levels. Fluorescence microscopy analysis of GFP-LC3 transfected cells and quantification of autophagosomes showed that the significant increase in the number of autophagosomes induced by culture in DM was inhibited by the autophagy inhibitor Asn or 3-MA (Figures 1F,G). Taken together, these results indicate that autophagy is essential for adipogenesis.

### Icariin suppresses adipogenesis by inhibiting autophagy

**Figure 2:**
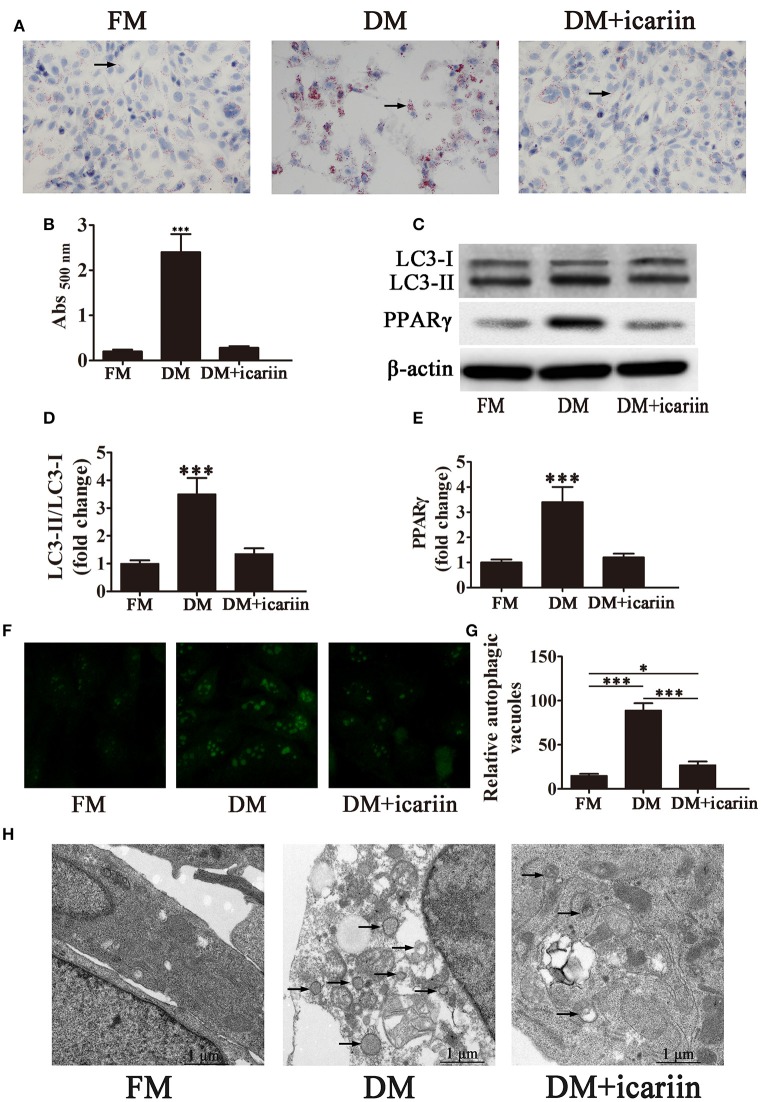
**Icariin treatment inhibits adipogenesis of 3T3-L1 cells by suppressing autophagy**. 3T3-L1 preadipocytes were incubated in adipocyte DM in the presence or absence of 5 μM icariin. **(A)** Oil Red O staining showing the effect of icariin on lipid droplet accumulation. **(B)** Quantification of intracellular lipids of 3T3-L1 cells by spectrophotometry. Data represent the mean ± *SD*. ^***^P < 0.001 vs. the untreated control (*n* = 5). **(C)** Western blot analysis the expression of the autophagy markers LC3-I, LC3-II, and PPARγ with β-actin as the loading control. **(D,E)** The LC3-II/LC3-I ratio and relative PPARγ expression were quantified by densitometric scanning and graphed. Data indicate the mean ± *SD* (*n* = 5). ^***^p < 0.001 vs. FM. **(F)** Representative fluorescence microscopy images of GFP-LC3 transfected cells treated as indicated (magnification: 400×). **(G)** The number of autophagosomes was counted in 10 random fields. The data represent the mean ± *SD*. (*n* = 10), ^*^P < 0.05, ^***^P < 0.001. **(H)** Electron microscopy images (5000×) show the inhibition of autophagy in response to icariin treatment. Arrows, autophagic vacuoles. FM, control medium; DM, differentiation medium.

Culture of 3T3-L1 preadipocytes in DM in the presence or absence of icariin showed that icariin inhibited adipogenesis, as illustrated by Oil Red O staining showing the suppression of lipid droplet formation by icariin (Figures 2A,B). Western blot assessment of LC3 showed that icariin restored the increase in the LC3-II:LC3-I ratio induced by DM in 3T3-L1 cells (Figures 2C,D). PPARγ expression was also inhibited in response to icariin treatment (Figure 2E). Assessment of autophagosome formation by GFP-LC3 fluorescence microscopy and quantification of autophagosomes showed that the significant increase in the number of autophagosomes induced by DM was inhibited by icariin treatment (Figures 2F,G). Electron microscopy was used to show the inhibition of autophagy by icariin, as seen by the reduced number of autophagic vacuoles in cells exposed to DM in the presence of icariin (Figure 2H). Taken together, these results indicate that icariin inhibits the differentiation of preadipocytes into adipocytes through the suppression of autophagy.

### Icariin inhibits autophagy and adipogenesis via the AMPK signaling pathway

**Figure 3:**
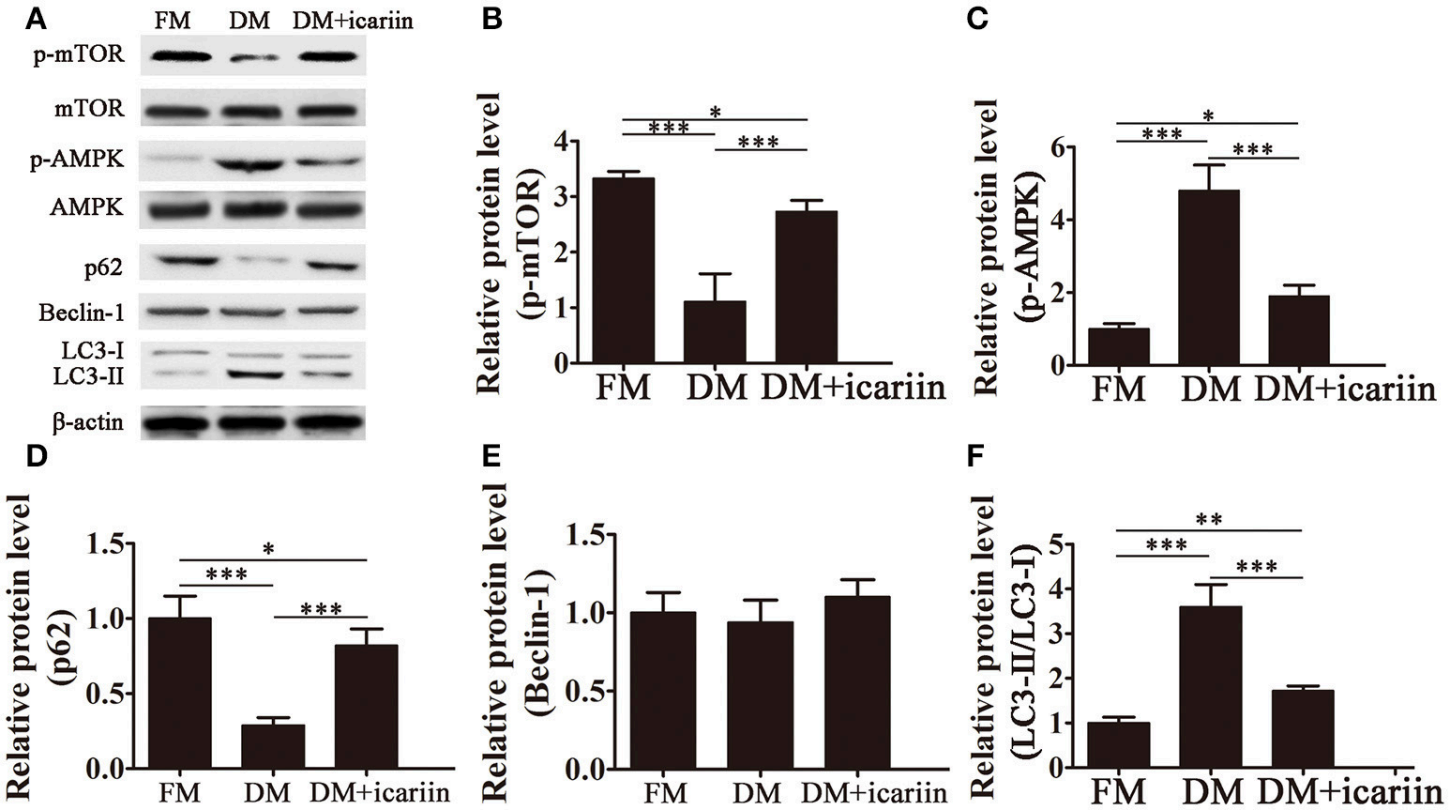
**Icariin suppressed autophagy by inhibiting AMPK activation**. 3T3-L1 preadipocytes were incubated in adipocyte DM in the presence or absence of 5 μM icariin. **(A)** The expression of autophagy related proteins and the activation of the AMPK/mTOR pathway were analyzed by western blotting. **(B–F)** The levels of phosphorylated mTOR **(B)** and AMPK **(C)** (normalized to the levels of mTOR and AMPK, respectively), and the expression of Beclin-1 **(E)**, p62 **(D)**, and LC3 **(F)** were determined by densitometric scanning and graphed. Data represent the mean ± *SD*. ^*^P < 0.05, ^**^P < 0.01, ^***^P < 0.001 (*n* = 5).

To further analyze the mechanisms underlying the effect of icariin, the activation of the AMPK pathway and the expression of autophagic markers were assessed by western blotting. The results showed that icariin significantly activation of mTOR and subsequently, inhibition of the phosphorylation of AMPK induced by culture of 3T3-L1 cells in DM (Figures 3A–C). Adipocyte differentiation was accompanied by a significant downregulation of p62, an adaptor protein for autophagy that binds to LC3, and a significant increase in the LC3-II:LC3-I ratio, while icariin partially restored p62 levels and inhibited the increase in the LC3-II:LC3-I ratio (Figures 3D,F). However, the levels of the autophagic marker Beclin-1 were not significantly affected by culture in DM or icariin treatment (Figure 3E). These results indicated that the effect of icariin on the inhibition of autophagy and adipogenesis may be mediated by the inhibition of AMPK signaling.

### Icariin inhibits orbital muscle adipose tissue expansion and lipid droplet accumulation in a mouse model of GO *In vivo*

**Figure 4:**
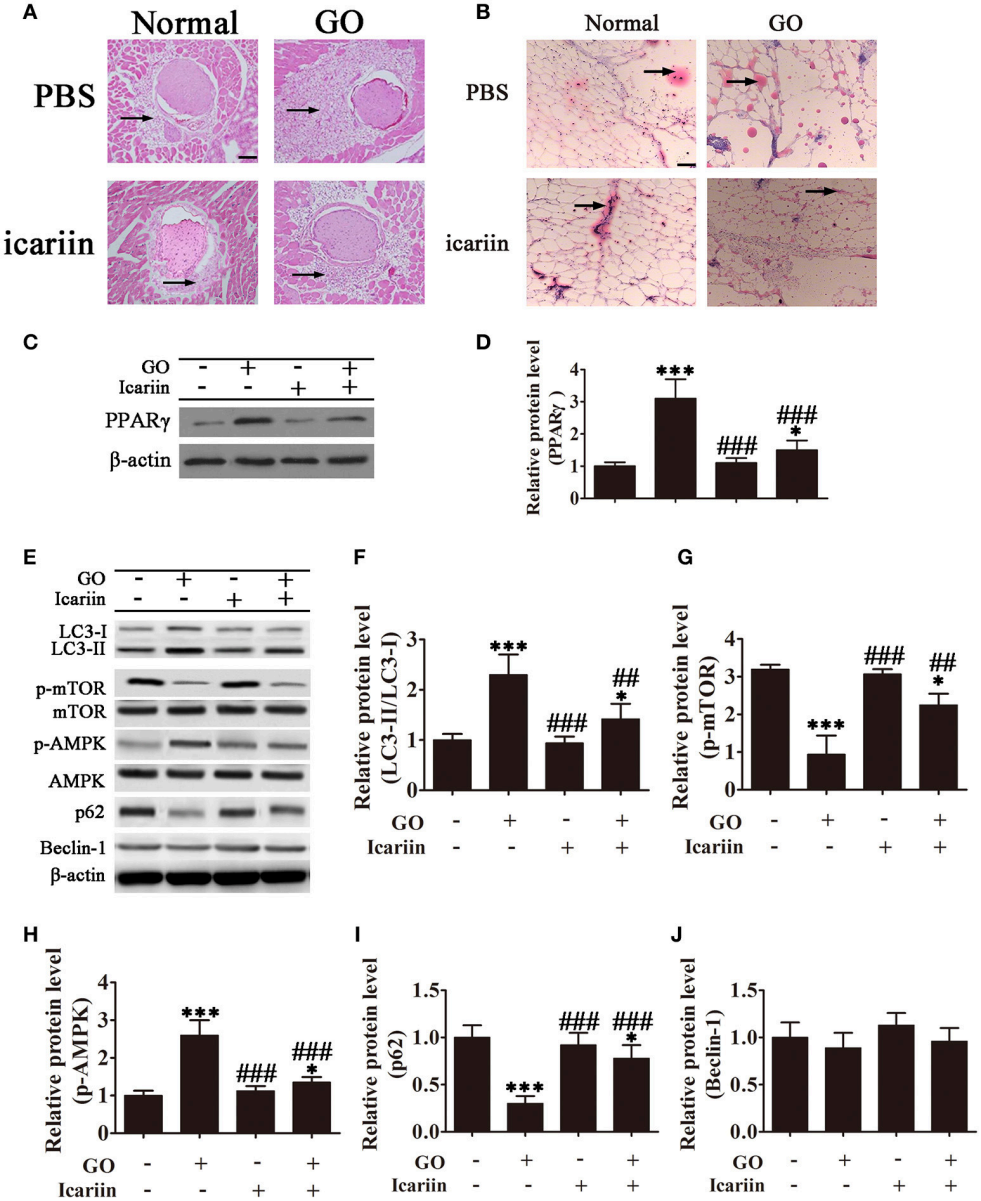
**Icariin inhibits the expansion of adipose tissue and lipid droplet accumulation in retrobulbar fat in a mouse model of Graves' orbitopathy (GO). (A)** Hematoxylin and eosin staining showing adipose tissue widely separating the orbital muscle fiber bundles in the GO group and the suppression of adipogenesis by icariin. Scale bar: 20 μm. **(B)** Oil Red O staining of orbital tissue sections showing icariin suppression of lipid droplet accumulation in TSHR induced GO mice. Scale bar: 20 μm. **(C)** Western blot analysis of the expression of the adipose differentiation marker PPARγ with β-actin as the loading control. **(D)** The relative PPARγ expression was quantified by densitometric scanning and graphed. Data indicate the mean ± SD (*n* = 5). ^*^P < 0.05, ^***^P < 0.001 vs. Normal control. ^###^*P* < 0.001 vs. GO group. **(E)** The expression of autophagy related proteins and the activation of the AMPK/mTOR pathway were analyzed by western blotting of retrobulbar adipose tissue lysates. **(F–J)** Densitometric quantification of western blot data in **(E)**. Data represent the mean ± *SD*. ^*^P < 0.05, ^**^P < 0.01, ^***^P < 0.001 vs. Normal control. ^##^*P* < 0.01, ^###^*P* < 0.001 vs. GO group. (*n* = 5).

The effect of icariin on adipogenesis was further examined *in vivo* in a mouse model of Ad-TSHR289 induced Graves' disease. Imaging of HE stained orbital sections showed the presence of adipose tissue separating orbital muscle fiber bundles in the GO group, indicating extensive adipogenesis, which was partially inhibited by icariin treatment (Figure 4A). Oil Red O staining showed that icariin inhibited lipid droplet formation in tissue sections from Ad-TSHR289 induced GO mice (Figure 4B). Western blot analysis showed that PPARγ expression was inhibited with icariin treatment (Figures 4C,D). Western blot analysis of orbital tissue homogenates showed a significant increase in the LC3-II:LC3-I ratio and a significant downregulation of p62 in GO mice, which were partially restored by icariin treatment (Figures 4E,F,I), whereas GO induction or icariin treatment had little effect on the levels of Beclin-1 (Figures 4E,J). Consistent with the *in vitro* findings, analysis of orbital tissues by western blotting showed that the levels of phospho-mTOR and phospho-AMPK were significantly increased in GO mice, whereas icariin treatment reversed this effect (Figures 4E,G,H). Taken together, these results indicated that icariin inhibits orbital muscle adipogenesis in a mouse model of GO, and this effect may be mediated by the suppression of autophagy through the AMPK signaling pathway.

## Discussion

The pathogenetic mechanisms leading to the development of GO, the most frequent extrathyroidal manifestation of Graves' disease, remain incompletely understood, making its therapeutic management difficult. GO is an inflammatory autoimmune disease that is thought to be triggered by recognition of the TSHR by autoreactive T-lymphocytes in the orbit (Iyer and Bahn, 2012). Crosstalk between TSHR and other factors and signaling molecules results in orbital autoimmune reactions leading to the proliferation of orbital fibroblasts, differentiation of preadipocytes into adipocytes, production of autoantibodies, secretion of cytokines, and infiltration of extraocular muscles (Smith, 2010). Improving our understanding of the mechanisms underlying the pathogenesis of GO could be of value for the design of effective therapies for this disease. Here, we examined the effect of icariin, a flavonoid extract used in traditional Chinese herbal medicine, on autophagy mediated adipogenesis in preadipocytes *in vitro* and in a mouse model of GO *in vivo* and explored the underlying mechanisms.

Inhibition of autophagy suppressed the differentiation of preadipocytes into adipocytes. The role of autophagy in the differentiation of orbital fibroblasts into mature adipocytes during the progression of GO has been described previously in a study that showed increased formation of autophagic vacuoles and upregulation of autophagy related genes in GO tissues and cells (Yoon et al., 2015). Studies have shown that autophagy is required for adipogenesis. Tissue-specific deletion of the autophagy genes *atg5* or *atg7* in white adipose tissue results in a phenotype characterized by reduced fat deposits and unusual morphology of adipocytes in mutant mice (Baerga et al., 2009; Singh et al., 2009; Zhang et al., 2009). These *in vivo* data were supported by *in vitro* studies using 3T3-L1 preadipocytes or mouse embryonic fibroblasts, in which genetic and pharmacological inhibition of autophagy blocked adipocyte differentiation.

In the present study, treatment of 3T3-L1 cells with icariin during culture in adipocyte DM inhibited adipogenesis, and western blot assessment of the LC3-II/LC3-I ratio suggested that this effect could be mediated by the inhibition of autophagy. Contrary to our results, icariin was shown to promote differentiation of cultured human osteoblasts by increasing the production of bone morphogenetic protein-2 (Yin et al., 2007). The protective effects of icariin were demonstrated in a study of H_2_O_2_ induced human umbilical vein endothelial cell injury, in which icariin protected cells against damage by downregulating caspase-3 and inhibiting apoptosis (Wang and Huang, 2005). Icariin has anticancer activity in a variety of tumors, including breast cancer, Burkitt lymphoma, and liver cancer (Zhang et al., 2013; Li W. et al., 2014; Li Z. J. et al., 2014), and in esophageal cancer, icariin exerts antitumor effects by modulating apoptosis (Fan et al., 2016). The antiapoptotic and anti-inflammatory effects of icariin were shown in a mouse model of cisplatin induced acute renal injury, in which icariin improved renal damage by downregulating tumor necrosis factor α, NF-κB, cleaved caspase-3, and Bax (Ma et al., 2015). In BM-EPCs, icariin exerts a cytoprotective effect against H_2_O_2_ induced injury by modulating both autophagy and apoptosis (Tang and Lane, 2012). Furthermore, the antiapoptotic and antiautophagic effects of icariin were shown to be mediated by restoration of mTOR activity. This was supported by our present results showing that icariin inhibited autophagy potentially by suppressing the activation of the AMPK/mTOR pathway. Western blot analysis showed that icariin inhibited the phosphorylation of AMPK and mTOR induced by incubation of preadipocytes in adipogenic induction medium. These effects occurred in parallel with the suppression of DM-induced autophagy, as shown by the restoration of the increased LC3-II:LC3-I ratio and downregulation of p62. These results were confirmed *in vivo* in a mouse model of GO, in which icariin suppressed adipose tissue deposition and lipid droplet accumulation in orbital tissues in GO mice concomitant with the inhibition of autophagy and reduction of AMPK/mTOR activation. Our results indicate that the protective effects of icariin mediated by the inhibition of adipogenesis in GO may be mediated by the modulation of AMPK-dependent autophagy. Further experiments are necessary to rule out the involvement of other mechanisms of cell death and other signaling pathways in the protective effects of icariin on GO.

In summary, we showed that icariin inhibits adipogenesis in preadipocytes *in vitro* and in a mouse model of GO *in vivo*, and these effects may be mediated by the suppression of autophagy through the modulation of the AMPK/mTOR pathway. Our results reveal a possible mechanism underlying the protective effects of icariin and identify icariin as a potential therapeutic agent for the treatment of GO.

## Author contributions

HL and YY conceived and designed the experiments. YZ and XZ performed the experiments. LG analyzed the data. RX contributed reagents/materials/analysis tools.

### Conflict of interest statement

The authors declare that the research was conducted in the absence of any commercial or financial relationships that could be construed as a potential conflict of interest.

## Supplementary material

Figure S1 **Effect of icariin on cell viability and apoptosis in 3T3-L1 cells. (A,B)** 3T3-L1 cells were treated with increasing concentrations of icariin (0, 1, 2.5, 5, and 10 μM) for 48 h. Cell viability was assessed by the MTT assay **(A)** and apoptosis was assessed by Annexin V/FITC staining and flow cytometry **(B)** to determine non-toxic concentrations of icariin. All values are presented as the mean ± *SD* (*n* = 5).

Figure S2 **Adipocyte differentiation from 3T3-L1 pre-adipocytes. (A,B)** 3T3-L1 cells were incubated in adipocyte DM for 1, 7, and 14 days. **(A)** Bright-field microscopic images of differentiating cells. **(B)** Lipid droplet accumulation was visualized by Oil Red O staining. **(C)** Quantification of intracellular lipids after incubation in DM for different times. Oil Red O stained lipids were extracted into isopropanol and the intensity of staining was determined by spectrophotometry. Data represent the mean ± *SD*. ^***^*P* < 0.001 vs. the untreated control (*n* = 5).
