# Supplementary figures and images for: Icariin Inhibits AMPK-Dependent Autophagy and Adipogenesis in Adipocytes In vitro and in a Model of Graves' Orbitopathy In vivo
Source: Front Physiol. 2017 Feb 13;8:45. doi: 10.3389/fphys.2017.00045 (PMC5303717; doi:10.3389/fphys.2017.00045)

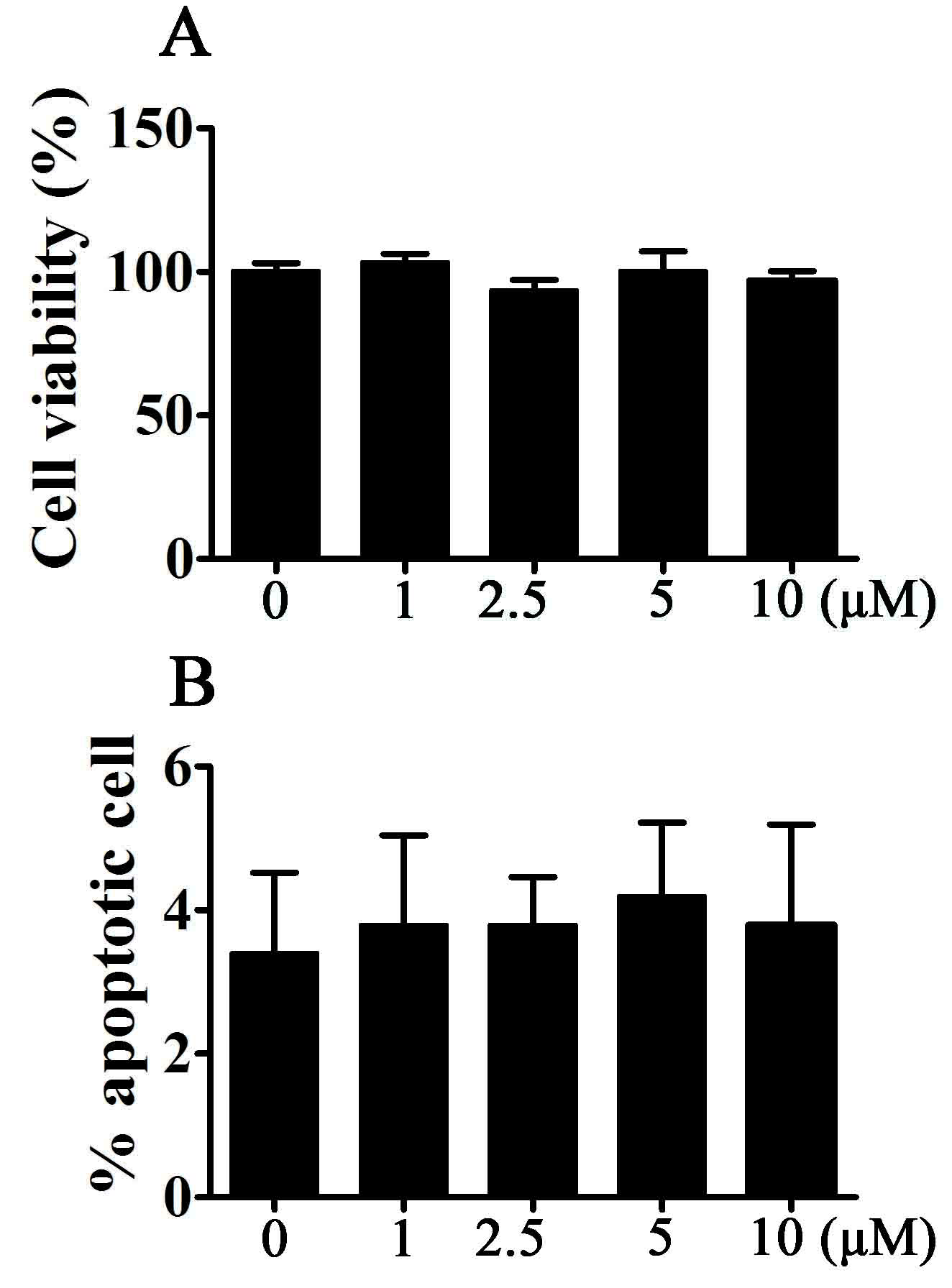

Supplement: Figure S1 — Effect of icariin on cell viability and apoptosis in 3T3-L1 cells. (A,B) 3T3-L1 cells were treated with increasing concentrations of icariin (0, 1, 2.5, 5, and 10 μM) for 48 h. Cell viability was assessed by the MTT assay (A) and apoptosis was assessed by Annexin V/FITC staining and flow cytometry (B) to determine non-toxic concentrations of icariin. All values are presented as the mean ± SD (n = 5). [file Image1.TIF]

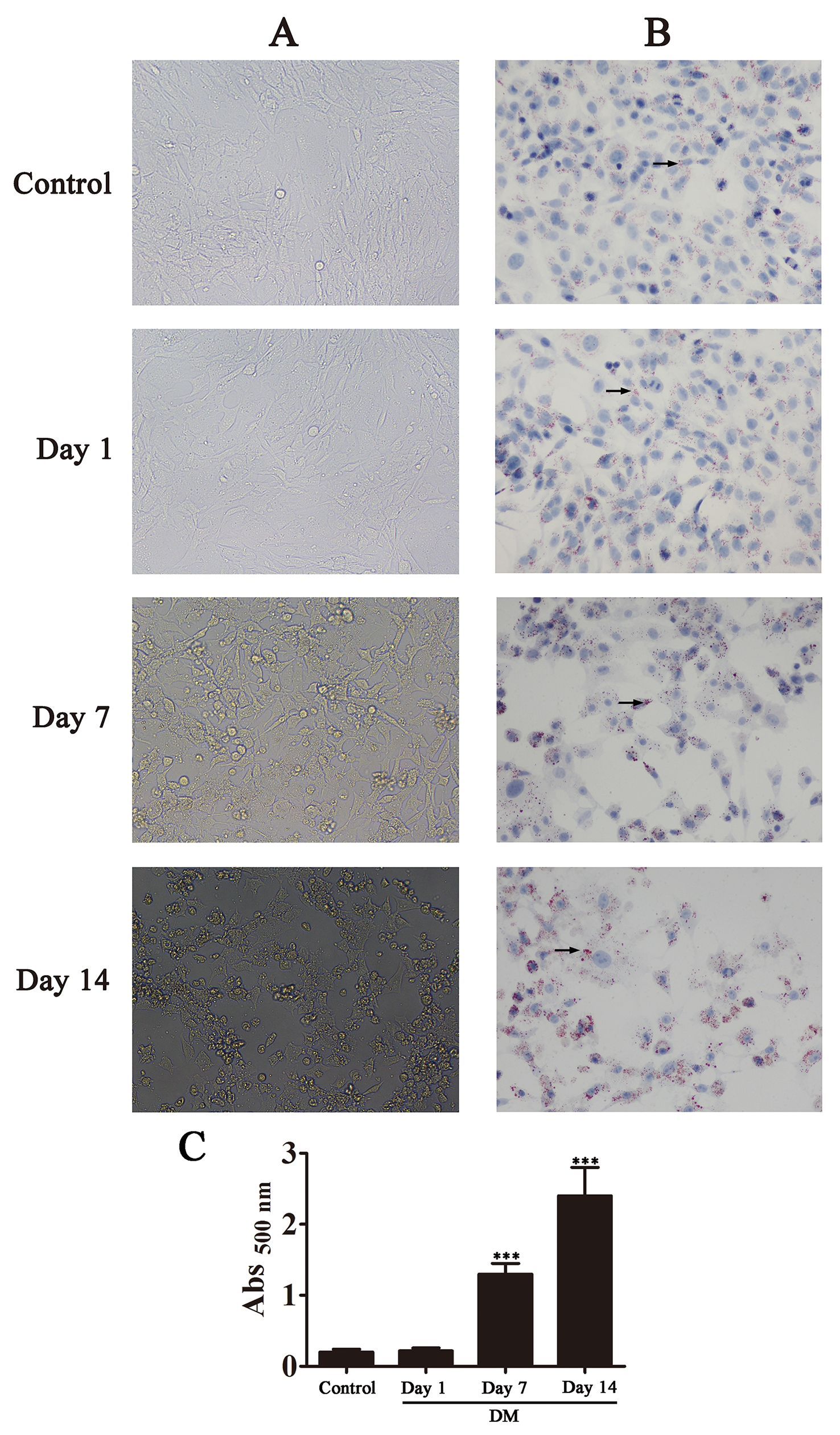

Supplement: Figure S2 — Adipocyte differentiation from 3T3-L1 pre-adipocytes. (A,B) 3T3-L1 cells were incubated in adipocyte DM for 1, 7, and 14 days. (A) Bright-field microscopic images of differentiating cells. (B) Lipid droplet accumulation was visualized by Oil Red O staining. (C) Quantification of intracellular lipids after incubation in DM for different times. Oil Red O stained lipids were extracted into isopropanol and the intensity of staining was determined by spectrophotometry. Data represent the mean ± SD. ***P < 0.001 vs. the untreated control (n = 5). [file Image2.TIF]
